# The changes of the peripheral CD4+ lymphocytes and inflammatory cytokines in Patients with COVID-19

**DOI:** 10.1371/journal.pone.0239532

**Published:** 2020-09-25

**Authors:** Hua-Bao Sun, Yi-Ming Zhang, Li-Gui Huang, Qi-Nan Lai, Qun Mo, Xin-Zhou Ye, Tao Wang, Zhong-Zhen Zhu, Xiao-Lin Lv, Yan-Ji Luo, Shi-Ding Gao, Jin-Song Xu, Hao-Hao Zhu, Ting Li, Zhan-Ke Wang

**Affiliations:** 1 Department of Clinical Laboratory, The Affiliated Infectious Diseases Hospital of Nanchang University, Nanchang, Jiangxi, China; 2 Department of Clinical Laboratory, The 908th Hospital of The Joint Logistics Support Force of The Chinese People’s Liberation Army, Nanchang, Jiangxi, China; 3 Department of Nosocomial infection, The 908th Hospital of The Joint Logistics Support Force of The Chinese People’s Liberation Army, Nanchang, Jiangxi, China; 4 Department of Clinical Laboratory, The First Affiliated Hospital of Nanchang University, Nanchang, Jiangxi, China; 5 Department of Clinical Laboratory, The Second Affiliated Hospital of Hunan Normal University, Changsha, Hunan, China; 6 Department of Infectious Disease, The 908th Hospital of The Joint Logistics Support Force of The Chinese People’s Liberation Army, Nanchang, Jiangxi, China; 7 Department of respiration, The 908th Hospital of The Joint Logistics Support Force of The Chinese People’s Liberation Army, Nanchang, Jiangxi, China; 8 School of Biotechnology, Jiangnan University, Wuxi, Jiangsu, China; Tulane National Primate Research Center, UNITED STATES

## Abstract

To investigate the clinical value of changes in the subtypes of peripheral blood lymphocytes and levels of inflammatory cytokines in patients with COVID-19, the total numbers of lymphocytes and CD4+ lymphocytes and the ratio of CD4+/CD8+ lymphocytes were calculated and observed in different groups of patients with COVID-19. The results show that the lymphocytopenia in patients with COVID-19 was mainly manifested by decreases in the CD4+ T lymphocyte number and the CD4+/CD8+ ratio. The decreased number of CD4+ T lymphocytes and the elevated levels of TNF-α and IL-6 were correlated with the severity of COVID-19 disease.

## Introduction

Novel coronavirus pneumonia (NCP) is mainly transmitted through respiratory droplets and close contact. The World Health Organization (WHO) lists NCP as a public health emergency of international concern [1] and officially named the disease caused by the novel coronavirus disease 2019 (COVID-19) [2]. Characteristic chest CT imaging patterns, positive nucleic acid detection in nasal and throat swab samples, normal or decreased numbers of peripheral white blood cells, decreased numbers of lymphocytes and increased levels of inflammatory cytokines are the key factors in the diagnosis of COVID-19 [3]. There are many kinds of lymphocytes in human peripheral blood, including CD3+CD4+ helper T lymphocytes (CD4+ T lymphocytes) and CD3+CD8+ cytotoxic T lymphocytes (CD8+ T lymphocytes). The percentage of CD4+ T lymphocytes and the ratio of CD4+/CD8+ lymphocytes are decreased in HIV-infected patients. To investigate whether the peripheral blood lymphocytopenia in COVID-19 patients was mainly caused by the decrease in CD4+ T lymphocytes, in this study, patients admitted to our hospital with different severities of COVID-19 were examined as subjects. The total number of lymphocytes, the percentages of lymphocyte subtypes and the levels of inflammatory cytokines (TNF-α and IL-6) secreted by CD4+ helper T lymphocytes in the peripheral blood were detected by hematology counter and flow cytometer, respectively. The number of CD4+ lymphocytes and the ratio of CD4+/CD8+ lymphocytes were calculated and observed, and a new method for studying the mechanism of 2019-nCoV-induced lymphocyte reduction was provided.

## Materials and methods

### Study participants

A total of 35 patients (26 men and 9 women; age range: 8–70 years) with COVID-19 who were admitted to The Affiliated Infectious Diseases Hospital of Nanchang University were evaluated. According to the diagnostic and clinical classification criteria of the Novel Coronavirus Infection Prevention and Control plan (2nd Edition) issued by the National Health Commission of the People's Republic of China, all the patients were divided into the general COVID-19 group (n = 13, 10 men and 3 women), severe COVID-19 group (n = 12, 9 men and 3 women) and critical COVID-19 group (n = 10, 8 men and 2 women). The normal control group (n = 20, 15 men and 5 women) included normal physical examination personnel. There were no significant differences in the ratio of men to women and age among the groups (p>0.05). The clinical symptoms of patients with COVID-19 include fever, cough, sore throat and muscle ache. The results of the 2019-nCoV nucleic acid tests performed on throat swabs were all positive in all the patient groups, and CT showed the changes characteristic of viral pneumonia. Laboratory examination showed that the white blood cell counts in the peripheral blood were normal or decreased, and the total number of lymphocytes was decreased. All the patients with COVID-19 had a history of travel to Wuhan, Hubei Province, or to an epidemic area outside Wuhan, Hubei Province. None of the patients died during the study, and the results of the 2019-nCoV nucleic acid tests in the normal control group were all negative. The study protocol was approved by the ethics committee of the Affiliated Infectious Diseases Hospital of Nanchang University (approval no. 202005, Nanchang, China), and written informed consent was obtained from all participants when the participants were awake or from their next of kin when the participants were minors or/and comatose. None of the patients or normal controls suffered from AIDS, influenza virus A or B infection, hepatitis B virus infection, tuberculosis infection, autoimmune diseases or other types of pneumonia.

### Detection of 2019-nCoV nucleic acids

Throat swabs from the patients were tested for the presence of 2019-nCoV nucleic acids by RT-PCR on an ABI7500 PCR amplification instrument (Applied Biosystems Inc., California, USA). The conserved gene sequences of open reading frame (ORF1a/1b) and nucleocapsid protein (N) in the 2019-nCoV genome were used as targets. When the ORF1a/1b and N target fragments were both positive at the same time, and the rising peaks were obvious, the nucleic acid test result was determined to be positive. The 2019-nCoV nucleic acid RT-PCR test kit (including the amplification primers) was produced by Shanghai BioGerm Biotechnology Co., Ltd (Shanghai). The National Machinery Registration number of the test kit is 20203400065. The catalog number of the RT-PCR test kit is ZC-HX-201-2.

### Detection of peripheral lymphocytes

The total numbers of peripheral white blood cells and lymphocytes in the patients were detected by impedance combined with laser on a BC-6900Plus automatic hematology analyzer (Shenzhen Mindray Biomedical Electronic Co., Ltd., Shenzhen, China). The detection reagent for peripheral blood cell analysis was purchased from Shenzhen Mindray Biomedical Electronic Co., Ltd (Shenzhen, China). The name of the cell staining solution used for blood cell analysis is M-60FN staining solution (Mindray), which product number is 105-012183-00; The name of hemolysin for hematology analysis is M-60LD hemolysin (Mindray), which product number is 105-012177-00; The name of the diluent used for blood cell analysis is DS diluent (Mindray), which product number is 105-005707-00. The blood cell control materials were produced by Beckman Coulter Co., Ltd. The name of Control materials of blood cells used for blood cell analysis is COULTER 5C CELL Control (Beckman Coulter), which product number is 7547001. The quality control data of the blood cell control materials were used as the control. The total number of lymphocytes was one of the results obtained by routine blood cell analysis.

### Detection of peripheral CD4+ T lymphocytes, CD8+ T lymphocytes and CD4+/CD8+ lymphocyte ratio

The detection of the subtypes of lymphocytes in the peripheral blood of the patients was analyzed on a Beckman Coulter DxFLEX flow cytometer (Suzhou Saijing Biotechnology Co., Ltd, Suzhou, China). The lymphocyte population was gated based on brightly positive CD45 staining and low SSC in the CD45 vs SSC dot plot. Then, the CD3 vs SSC dot plot shows the lymphocyte population, and the T lymphocyte population was gated based on brightly positive CD3 staining. Then, suppressor/cytotoxic (CD3+CD8+) and helper/inducer (CD3+CD4+) lymphocytes were identified in the CD8 vs CD3 dot plot and the CD4 vs CD3 dot plot, respectively. Ten microliters of anti-CD molecule antibodies labeled with different fluorescent dyes and 50 μl of EDTA anticoagulant-treated peripheral blood were fully mixed, protected from light, and incubated at room temperature (19–25°C) for 15–20 minutes. Then, 500 µl of hemolysin(Beckman) was added to the solution, and the solution was vortexed for 10 seconds, mixed well, protected from light, and incubated at room temperature (19–22°C) for 15–20 minutes until the solution changed from turbid to clear. Then, 200 µl of saline was added, and the solution was mixed well for detection by flow cytometry. The test kits used to analyze the lymphocyte subtypes, including anti-CD molecule antibodies labeled with different fluorescent dyes, were produced by Beckman Coulter Co., Ltd. (740 West 83rd St., Hialeah, FL 33014, USA). The production approval document number (China) for the lymphocyte subtype test kits was guoxiezhujin20173401372. The medical device production license number (China) of the BECKMAN COULTER DxFLEX Flow cytometer is sushiyaojianxieshenchanxu20130069. The catalog number of the test kits named CYTO-STAT tetra CHROME for detection of lymphocyte subtype(Beckman Coulter Co., Ltd), which including CD45, CD3, CD4 and CD8 antibodies is 6607013. The name of hemolysin reagent is Opti Lyse C(Beckman), which catalog number is A11895. All antibodies were undiluted for use in this study.

The absolute number of CD4+ T lymphocytes was calculated as the total number of lymphocytes × the percentage of CD4+ T lymphocytes. The total number of lymphocytes in the patients was determined from the results of routine blood cell analysis.

### Detection of the plasma levels of TNF-α and IL-6

The levels of TNF-α and IL-6 in the plasma of the patients were simultaneously determined by cytometric bead array (CBA) via flow cytometry. Then, 3–5 mL of heparin anticoagulant-treated blood from the patients was centrifuged to obtain plasma. Twenty-five microliters of immune microspheres and 25.0 μL of patient plasma were mixed and incubated in the dark at room temperature for 2.5 hours. Then, 25 μL fluorescent detection reagent was added. After washing the microspheres, the fluorescence intensity of the washed microspheres was quantitatively measured by a Beckman Coulter DxFLEX flow cytometer (Suzhou, China). Different concentrations of TNF-α and IL-6 standards were analyzed together with the plasma to be tested. The fluorescence intensity of the microspheres was determined by flow cytometry, and the fluorescence intensity was in direct proportion to the concentrations of TNF-α and IL-6 examined. The test kit used for the detection of TNF-α and IL-6 in plasma by CBA was produced by Jiangxi Saiji Biotechnology Co., Ltd. (Nanchang, China), which national machinery registration number is 20180010. The human plasma TNF-α and IL-6 detection kit is named human Th1 and Th2 subgroup detection kit(Jiangxi Saiji), which product number is P010001. The antibodies of TNF-α and IL-6 were undiluted for use in this study.

### Statistical analysis

All the data are expressed as the mean ± standard deviation (SD). Comparison of continuous variables between two groups was performed using Student’s t-test. P < 0.05 was considered to be statistically significant. All the statistical analyses were performed using IBM SPSS Statistics 23.0 software for Windows (SPSS Inc., Chicago, IL, USA) on a computer.

## Results

### Changes in the numbers of peripheral blood lymphocytes and CD4+ T lymphocytes and the ratio of CD4+/CD8+

**Fig 1:**
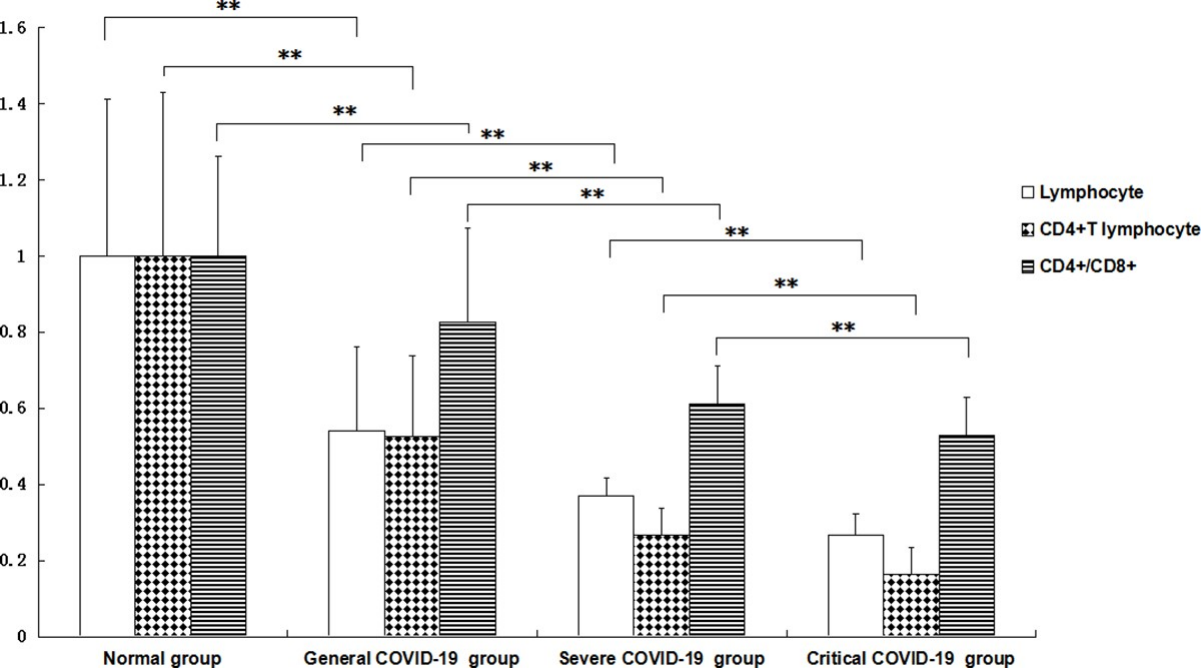
Changes in the total numbers of lymphocytes and CD4+ lymphocytes and the ratio of CD4+/CD8+ lymphocytes in the peripheral blood of patients with different severities of COVID-19. The total numbers of lymphocytes and CD4+ lymphocytes and the ratio of CD4+/CD8+ lymphocytes in the patients in the general COVID-19 group were significantly lower than those in the normal control group. The levels in the severe COVID-19 group were significantly lower than those in the general COVID-19 group, and the levels in the critical COVID-19 group were significantly lower than those in the severe COVID-19 group. The total number of lymphocytes (10^9^ cells/L) and CD4+ lymphocytes (cells/µL) and the ratio of CD4+/CD8+ lymphocytes of the patients in the normal control group were all set to 1.0. ** p<0.01.

In the COVID-19 patients in the general, severe and critical groups, the numbers of peripheral lymphocytes and CD4+ T lymphocytes and the ratio of CD4+/CD8+ lymphocytes were significantly lower than those in the normal control group. The levels of peripheral lymphocytes and CD4+ T lymphocytes and the ratio of CD4+/CD8+ lymphocytes in the general group were lower than those in the normal control group (p = 0.000441252, 0.000404213, and 0.003613912, respectively, all <0.01), and the levels in the severe group were lower than those in the general group (p = 0.009585116, 0.000294487, and 0.004389093, respectively, all <0.01). The levels in the critical group were lower than those in the severe group (p = 0.00011258, 0.001189364, and 0.00764968, respectively, all <0.01). All the differences were significant, with *P*<0.01 (Fig 1).

### Correlation analysis between the number of peripheral blood lymphocytes and the number of CD4+ T lymphocytes

The correlation coefficient of the number of peripheral blood CD4+ T lymphocytes and the number of total lymphocytes in the 35 COVID-19 patients was 0.9051 (p = 0.000002, <0.01). In the patients with COVID-19, the decreased number of CD4+ T lymphocytes was positively correlated with the decreased number of total lymphocytes. The lower the number of CD4+ T lymphocytes was, the lower the number of total lymphocytes was. In the patients with COVID-19, the total number of peripheral blood lymphocytes decreased mainly as the number of CD4+ T lymphocytes decreased.

### Changes in the number of peripheral blood CD8+ T lymphocytes

**Fig 2:**
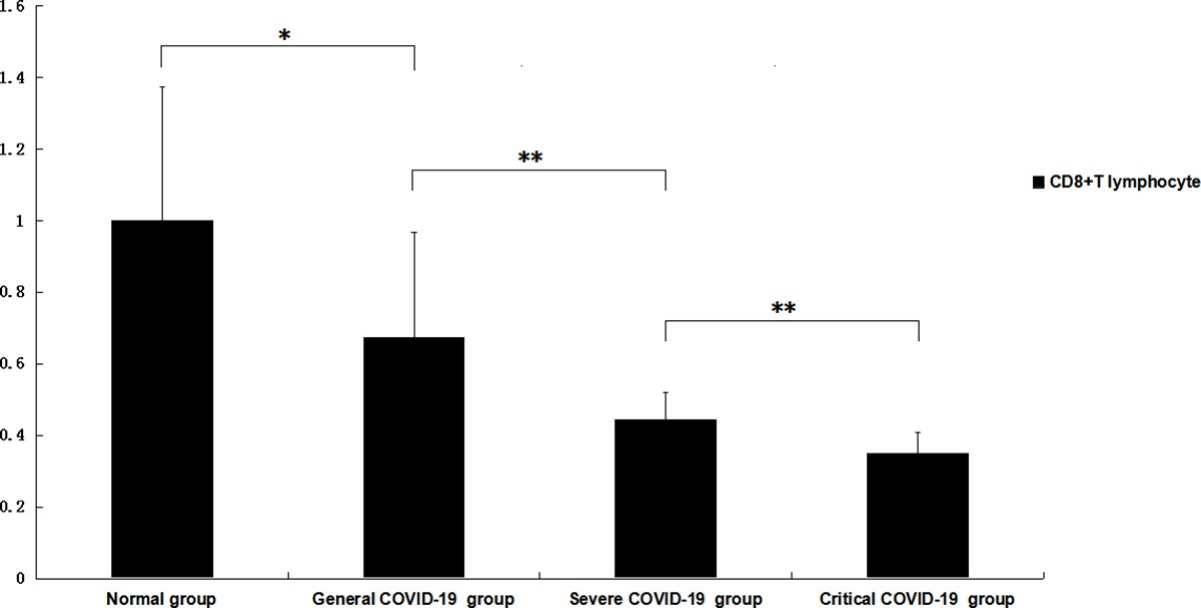
Changes in the number of CD8+ lymphocytes in the peripheral blood of patients with different severities of COVID-19. The total number of CD8+ lymphocytes in the patients in the general COVID-19 group was significantly lower than that in the normal control group, the levels in the severe COVID-19 group were significantly lower than those in the general COVID-19 group, and the levels in the critical COVID-19 group were significantly lower than those in the severe COVID-19 group. The total number of lymphocytes (10^9^ cells/L) and CD4+ lymphocytes (cells/µL) and the ratio of CD4+/CD8+ lymphocytes of the patients in the normal control group were all set to 1.0. * p<0.05; ** p<0.01.

The levels of peripheral CD8+ T lymphocytes in the general group were lower than those in the normal control group (p = 0.02, <0.05), and the levels in the severe group were lower than those in the general group (p = 0.007, <0.01). The levels in the critical group were lower than those in the severe group (p = 0.002, <0.01). All the differences were significant, with p<0.05 (Fig 2).

### Changes in the TNF-α and IL-6 levels in plasma

**Fig 3:**
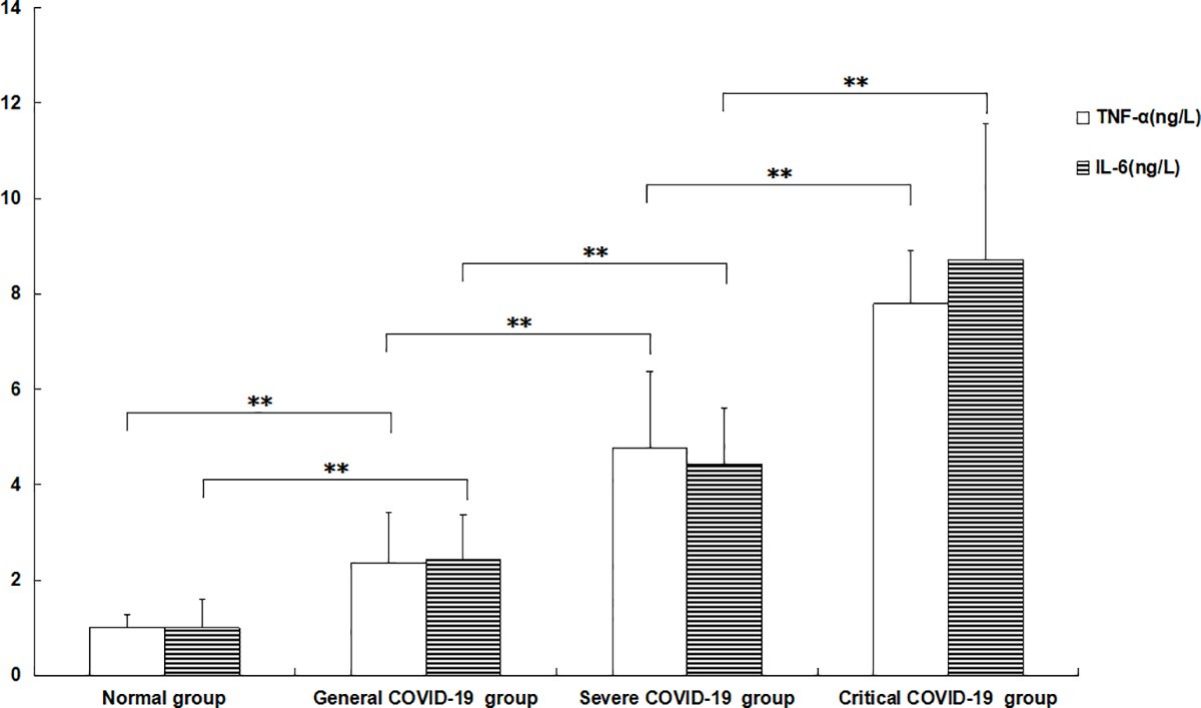
Changes in TNF-α and IL-6 levels in the peripheral blood of patients with different severities of COVID-19. The TNF-α and IL-6 levels of the patients in the general COVID-19 group were significantly higher than those in the normal control group. The levels in the severe COVID-19 group were significantly higher than those in the general COVID-19 group, and the levels in the critical COVID-19 group were significantly higher than those in the severe COVID-19 group. The TNF-α and IL-6 levels in the peripheral blood of patients in the normal control group were all set to 1.0. ** p<0.01.

The TNF-α and IL-6 levels in the peripheral blood of COVID-19 patients in the general, severe and critical groups were significantly higher than those in peripheral blood of the subjects in the normal control group. The levels of TNF-α and IL-6 in the general group were higher than those in the normal control group (p = 0.00000209 and 0.00001729, respectively), and the levels in the severe group were higher than those in the general group (p = 0.00007539 and 0.00004138, respectively). The levels in the critical group were higher than those in the severe group (p = 0.00003602 and 0.00006652, respectively). All the differences were significant, with *P*<0.01 (Fig 3).

## Discussion

Coronaviruses (CoV) are divided into four genera, including α−/β−/γ−/δ-CoV. The 2019-nCoV that causes COVID-19 is a β-coronavirus, which is an enveloped, positive single-stranded RNA (ssRNA) coronavirus. The shape of 2019-nCoV is round or oval with a diameter of 60–140 nm, as observed by electron microscopy. The human-to-human transmission of 2019-nCoV is clear. It was found that the genomic sequence of 2019-nCoV shares more than 85% identity with that of SARS-CoV [4]. ACE2 (angiotensin-converting enzyme 2), which is expressed in the lower respiratory tract of humans, is confirmed as a cellular receptor for 2019-nCoV [5]; this receptor mediates cellular entry of the virus and causes severe and potentially fatal respiratory tract infections. The incubation period is 1–14 days, mostly 3–7 days. The main clinical manifestations are fever, headache, dry cough, fatigue and muscle ache. Some patients have symptoms such as nasal congestion, pharyngeal pain and diarrhea. The clinical types of COVID-19 include the general type, severe type and critical type. Patients with severe and critical conditions can rapidly develop respiratory failure, septic shock and multiple organ failure after approximately 1 week [6]. All the patients in our study exhibited typical clinical manifestations, such as fever, cough, sore throat and muscle ache. Critical patients require mechanical ventilation with a ventilator, and severe patients have dyspnea, along with the characteristics of severe viral pneumonia observed on CT images. The clinical symptoms of general type patients are mild, and the lung CT showed characteristic images of mild viral pneumonia. Pulmonary CT imaging can diagnose viral pneumonia, but determining the cause of the disease requires 2019-nCoV nucleic acid detection. All of our subjects were positive according to a throat swab 2019-nCoV nucleic acid test, suggesting that the diagnosis of COVID-19 was clear and that there was no misdiagnosis.

It was reported that the total number of peripheral blood (PB) lymphocytes in patients with COVID-19 is decreased [7]. To study which type of lymphocyte exhibits the greatest decrease, we analyzed the lymphocyte subtypes in the PB of patients with COVID-19. The total number of lymphocytes was tested by routine blood tests, the percentage of lymphocyte subtypes was determined by flow cytometry, and the absolute number of lymphocytes in each subtype was calculated. The results showed that the lymphocytopenia in patients with COVID-19 was mainly manifested by the decrease in CD4+ helper T lymphocytes (the absolute number); in addition, the ratio of CD4+/CD8+ lymphocytes decreased, which showed a typical cellular immune dysfunction similar to that observed in HIV patients. A CD4+ T lymphocyte count below 200 cells/µL in the peripheral blood is the basis for antiviral treatment in HIV patients. Although there is no direct relationship between the genomes of the two viruses [8], the results of this study suggest that for COVID-19 patients with decreased CD4+ T lymphocyte numbers in their peripheral blood, the protease inhibitors used to treat human immunodeficiency virus (HIV) infection, such as lopinavir and ritonavir, are also worth trying and studying to improve the outcome of patients with COVID-19.

CD4+ T lymphocytes are mainly helper T lymphocytes (Th) and include Th1 and Th2, which can secrete inflammatory cytokines. At present, the specific mechanism by which CD4+ T lymphocyte numbers decrease in the peripheral blood of patients infected with SARS-CoV-2 is unclear. There are 4 possible mechanisms. (1) 2019-nCoV directly damages CD4+ T lymphocytes but does not invade CD4+ T lymphocytes. (2) 2019-nCoV directly invades CD4+ T lymphocytes and takes CD4+ T lymphocytes as host cells. (3) Patients with COVID-19 develop viremia and progress to systemic inflammatory response syndrome (SIRS). CD4+ T lymphocytes or other immune cells secrete a large amount of inflammatory cytokines, resulting in excessive consumption of CD4+ T lymphocytes. (4) SARS-CoV-2 inhibits the differentiation and production of CD4+ T lymphocytes. At present, there is no evidence that 2019-nCoV can directly invade peripheral blood CD4+ T lymphocytes, and there is no evidence that a 2019-nCoV receptor exists on the CD4+ T lymphocyte membrane. The maturation and formation of CD4+ T lymphocytes depend on the function of human thymus tissue cells, which can express the ACE2 receptor [9], suggesting that the damage to human thymus cells caused by 2019-nCoV may be a cause of the decrease in peripheral blood lymphocytes in patients with COVID-19.

The inflammatory cytokine storm plays an important role in the development of COVID-19 in patients [10]. TNF-α and IL-6 are important inflammatory cytokines, and we found that the levels of TNF-α and IL-6 in the plasma of patients with COVID-19 were significantly increased and positively correlated with the severity of the disease. The significant increase in the levels of TNF-α and IL-6 in the plasma of patients with COVID-19 is related to many kinds of inflammatory immune cells that secrete inflammatory factors. The secretion of inflammatory factors may require the assistance of CD4+ cells, which can secrete a large amount of inflammatory cytokines themselves. Our results suggest that the decrease in the number of CD4+ T lymphocytes in patients with COVID-19 may be related to the excessive consumption of CD4+ T lymphocytes. How to effectively increase the number of CD4+ T lymphocytes in the peripheral blood of patients with COVID-19 still requires further study.

We found that the CD4+ T cells in the peripheral blood were decreased in individuals infected with COVID-19 but that the levels of TNF-α and IL-6 in the plasma, which can be produced by CD4+ T cells, were significantly increased. The reason may be that in addition to CD4+ Th cells in the peripheral blood, a large number of monocytes in the blood and macrophages in human tissues, including the liver and intestine, can secrete large amounts of TNF-α and IL-6 into the blood in humans. The number of CD4+ Th cells in the peripheral blood decreased; however, this decrease does not mean that the abilities single CD4+ Th cells to secrete TNF-α and IL-6 decreased.

The number of CD4+ Th cells in the peripheral blood significantly decreased, but the plasma levels of inflammatory cytokines significantly increased in patients with COVID-19; this finding may be related to the increased secretion of TNF-α and IL-6 by a large number of immune cells other than CD4+ Th cells in the peripheral blood or the enhanced ability of single CD4+ Th cells to secrete TNF-α and IL-6 during 2019-nCoV infection. We detected a decrease in the number of CD4+ Th cells in the peripheral blood, but this decrease does not mean that the number of CD4+ Th cells in human tissues, such as lymph nodes or spleen, has decreased. The number of CD4+ Th cells in the peripheral blood significantly decreased, but the plasma levels of inflammatory cytokines significantly increased in patients with COVID-19, which may also be related to the fact that CD4+ T cells are sequestered in tissues and therefore are not detected in the blood.

The targeting of thymus tissue by 2019-nCoV may be an important reason for the decrease in CD4+ Th cells in the peripheral blood of COVID-19 patients. Many immune cells in the blood secrete TNF-α and IL-6, especially during infection and inflammation, and monocytes and macrophages in the peripheral blood and tissues mainly secrete large amounts of TNF-α and IL-6. This phenomenon is also the main reason why although the number of CD4+ Th cells in the peripheral blood significantly decreased, the plasma levels of TNF-α and IL-6 significantly increased in patients with COVID-19. The secretion of TNF-α and IL-6 by CD4+ Th cells in the peripheral blood may not be the main reason for the significant increase in TNF-α and IL-6 in the peripheral blood of COVID-19 patients.

Our study also found that not only the absolute number of CD4+ Th cells but also the absolute number of CD8+ Tc cells decreased in the peripheral blood. However, the decrease in the number of CD8+ Tc cells is not as substantial as that of CD4+ Th cells, resulting in a trend of decreasing ratios of CD4+/CD8+ lymphocytes in the peripheral blood with the severity of the disease. CD8+ Tc cells are cytotoxic T lymphocytes and belong to one of the subtypes of T lymphocytes. The differentiation and maturation of all T lymphocytes in the body, including CD4+ and CD8+ T lymphocytes, is dependent on thymocytes; therefore the targeting of thymus tissue by 2019-nCoV may be an important reason for the decrease in the number of CD8+ Tc cells in the peripheral blood of COVID-19 patients. Further study is needed to investigate the mechanism by which CD4+ and CD8+ T cell numbers decrease in the peripheral blood of COVID-19 patients.

## Conclusions

In patients with COVID-19, the number of peripheral blood lymphocytes decreased, mainly manifesting as a decrease in the number of CD4+ T lymphocytes, a decrease in the ratio of CD4+/CD8+ lymphocytes, and a decrease in the number of CD8+ lymphocytes; the degrees of these reductions was significantly correlated with the severity of disease. The levels of TNF-α and IL-6 in the peripheral blood were significantly increased in COVID-19 patients, the degree of this elevation was significantly correlated with the severity of disease. The significantly decreased levels of CD4+ T lymphocytes and ratio of CD4+/CD8+ lymphocytes and the significantly increased levels of TNF-α and IL-6 in the peripheral blood can be used as important laboratory indicators to assess the severity of COVID-19 in patients.

## Supporting information

S1 Table The experimental data of the numbers of lymphocytes, CD4+ T lymphocytes, and CD8+ lymphocytes and the ratio of CD4+/CD8+ lymphocytes in the peripheral blood of patients with different severities of COVID-19. Compared with the normal control group, * p<0.05, ** p < 0.01.
(DOC)

S2 Table The experimental data of the levels of TNF-α and IL-6 in the peripheral blood of patients with different severities of COVID-19. Compared with the normal control group, ** p < 0.01.
(DOC)
