# Supplementary material for: The changes of the peripheral CD4+ lymphocytes and inflammatory cytokines in Patients with COVID-19
Source: PLoS One. 2020 Sep 25;15(9):e0239532. doi: 10.1371/journal.pone.0239532 (PMC7518571; doi:10.1371/journal.pone.0239532)
Supplement: S1 Table — Compared with the normal control group, * p<0.05, ** p < 0.01. (DOC) [file pone.0239532.s001.doc]

**Table 1: The experimental data of the Lymphocyte，CD4+T lymphocyte, CD8+ lymphocyte，and ratio of CD4+/CD8+ in peripheral blood of patients with different severity of COVID-19.**

| Group | Lymphocyte（×10^9^） | CD4+T lymphocyte（Cell/µL） | CD8+ lymphocyte（Cell/µL） | CD4+/CD8+ |
| --- | --- | --- | --- | --- |
| **Normal control** | **2.11±0.87** | **1097±473** | **729±273** | **1.49±0.39** |
| **General COVID-19** | **1.14±0.47**** | **576±234**** | **492±214*** | **1.23±0.37**** |
| **Severe COVID-19** | **0.78±0.10**** | **293±76**** | **323±56**** | **0.91±0.15**** |
| **Critical COVID-19** | **0.56±0.12**** | **180±77**** | **255±42**** | **0.69±0.22**** |

**Vs** Normal Group * p<0.05, ** p<0.01
