# Supplementary material for: The changes of the peripheral CD4+ lymphocytes and inflammatory cytokines in Patients with COVID-19
Source: PLoS One. 2020 Sep 25;15(9):e0239532. doi: 10.1371/journal.pone.0239532 (PMC7518571; doi:10.1371/journal.pone.0239532)
Supplement: S2 Table — Compared with the normal control group, ** p < 0.01. (DOC) [file pone.0239532.s002.doc]

**Table 2: The experimental data of the TNF-α and Il-6 in peripheral blood of patients with different severity of COVID-19.**

| **Group** | **TNF-α(ug/L)** | **Il-6(ug/L)** |
| --- | --- | --- |
| **Normal control** | **4.39±1.18** | **3.11±1.84** |
| **General COVID-19** | **10.41±4.63**** | **7.61±2.84**** |
| **Severe COVID-19** | **21.0±6.99**** | **13.8±3.68**** |
| **Critical COVID-19** | **34.2±4.93**** | **27.08±8.86**** |

**Vs N**ormal group ** p<0.01
